# Promotion of the growth and yield of *Zea mays* by synthetic microbial communities from Jala maize

**DOI:** 10.3389/fmicb.2023.1167839

**Published:** 2023-05-19

**Authors:** Esaú De la Vega-Camarillo, Josimar Sotelo-Aguilar, Bibiana Rios-Galicia, Yuridia Mercado-Flores, Ramón Arteaga-Garibay, Lourdes Villa-Tanaca, César Hernández-Rodríguez

**Affiliations:** ^1^Laboratorio de Biología Molecular de Bacterias y Levaduras, Departamento de Microbiología, Escuela Nacional de Ciencias Biológicas, Instituto Politécnico Nacional, Ciudad de México, Mexico; ^2^Laboratorio de Aprovechamiento Integral de Recursos Bióticos, Universidad Politécnica de Pachuca, Hidalgo, Mexico; ^3^Laboratorio de Recursos Genéticos Microbianos, Centro Nacional de Recursos Genéticos, INIFAP, Jalisco, Mexico

**Keywords:** endophytic bacteria, plant growth-promoting bacteria (PGPB), Jala maize, synthetic microbial communities (SynCom), plant-microbe interaction, induced systemic resistance (ISR), biocontrol

## Abstract

Plant growth-promoting bacteria (PGPB) are a source of nutrient supply, stimulate plant growth, and even act in the biocontrol of phytopathogens. However, these phenotypic traits have rarely been explored in culturable bacteria from native maize landraces. In this study, synthetic microbial communities (SynCom) were assembled with a set of PGPB isolated from the Jala maize landrace, some of them with additional abilities for the biocontrol of phytopathogenic fungi and the stimulation of plant-induced systemic resistance (ISR). Three SynCom were designed considering the phenotypic traits of bacterial strains, including *Achromobacter xylosoxidans* Z2K8, *Burkholderia* sp. Z1AL11, *Klebsiella variicola* R3J3HD7, *Kosakonia pseudosacchari* Z2WD1, *Pantoea ananatis* E2HD8, *Pantoea* sp. E2AD2, *Phytobacter diazotrophicus* Z2WL1, *Pseudomonas protegens* E1BL2, and *P. protegens* E2HL9. Plant growth promotion in gnotobiotic and greenhouse seedlings assays was performed with Conejo landrace; meanwhile, open field tests were carried out on hybrid CPL9105W maize. In all experimental models, a significant promotion of plant growth was observed. In gnotobiotic assays, the roots and shoot length of the maize seedlings increased 4.2 and 3.0 times, respectively, compared to the untreated control. Similarly, the sizes and weights of the roots and shoots of the plants increased significantly in the greenhouse assays. In the open field assay performed with hybrid CPL9105W maize, the yield increased from 11 tons/ha for the control to 16 tons/ha inoculated with SynCom 3. In addition, the incidence of rust fungal infections decreased significantly from 12.5% in the control to 8% in the treatment with SynCom 3. All SynCom designs promoted the growth of maize in all assays. However, SynCom 3 formulated with *A. xylosoxidans* Z2K8, *Burkholderia* sp. Z1AL11, *K. variicola* R3J3HD7, *P. ananatis* E2HD8, *P. diazotrophicus* Z2WL1, and *P. protegens* E1BL2 displayed the best results for promoting plant growth, their yield, and the inhibition of fungal rust. This study demonstrated the biotechnological eco-friendly plant growth-promoting potential of SynCom assemblies with culturable bacteria from native maize landraces for more sustainable and economic agriculture.

## 1. Introduction

Plants and their microbiome are holobionts that coevolve modulating or enhancing the adaptation to the environment, fitness, competitivity, phytopathogen resistance, abiotic stress tolerance, health, and productivity of plants in natural or agricultural ecosystems (Chiu and Gilbert, 2015; Berg et al., 2021; Cesaro et al., 2021). Abundant studies on plant–bacteria interactions are available in the scientific literature, and their potential biotechnological applications to agriculture as bioinoculants have a significant market share (Nehra and Choudhary, 2015; Owen et al., 2015; Saritha and Tollamadugu, 2019; Nosheen et al., 2021). Plant–bacteria holobiont interactions are extraordinarily complex because the flow of metabolites between both components is bidirectional (Selosse et al., 2004; Wani et al., 2015; Sánchez-Cañizares et al., 2017; Hawkes et al., 2020). Bioinoculants and synthetic microbial communities (SynCom) contain microbial species that can synthesize several plant growth-promoting (PGP) and biopesticide metabolites (Bashan, 1998; Johnsen et al., 2001; Cavali et al., 2010; Souza et al., 2020; Kaur et al., 2022; Prigigallo et al., 2022; Shayanthan et al., 2022). The expression of these synergistic activities by specialized microbial species can be continued or alternated to maintain a constant rate of metabolite incorporation even under different environmental conditions and/or stages of the plant life cycle (Maier et al., 2009; Rana et al., 2011; Babu et al., 2015; Emami et al., 2019; Magallon-Servin et al., 2020; Hett et al., 2022).

The main source for bioinoculants or SynCom is the culturable fraction of autochthonous bacteria from the endosphere or rhizosphere of agricultural plants. However, the selection pressures derived from the use of abundant artificial fertilizers, pesticides, improved and genetically homogeneous varieties of plants, and antibiotics and antifungals used to control phytopathogens in seeds may have contributed to the loss of alpha diversity of plant growth-promoting bacteria (PGPB) in the endosphere of domesticated plants used in intensive agriculture (Lynch et al., 2004; Hussain et al., 2009; Zaller et al., 2016; Berg et al., 2017; Ohore et al., 2022). This decrease in microbial biodiversity in the endosphere of commercial plants has some implications for losing their tolerance to abiotic and biotic stress (Gutierrez and Grillo, 2022). This diversity loss has previously been observed in chicory roots (Verdin et al., 2006), soybeans (Vieira et al., 2007), and some legumes (Fox et al., 2007). Futhermore, the diversity of the seed microbiota of several cereal crops and rice has generally been shown to be higher in cultivated cereals than in wild ancestors, while more microbe–microbe interactions have been detected in wild relative species (Kim et al., 2020; Abdullaeva et al., 2021). In any case, the diversity of the endophytic microorganisms of plants used in traditional agriculture with wild, native, and ancestral plants helps maintain a potential pool of novel plant growth-promoting microorganism strains used in sustainable agriculture (Vibha and Neelam, 2012; Berg et al., 2017).

Maize (*Zea mays*) is the most produced and consumed grain globally, moreover, having the largest planting area worldwide. Maize is a primary consumer product in numerous countries (International Grains Council, 2022un). The enormous world demand for this cereal requires an increase in the area of available arable land as well as the use of all available biotechnological resources such as fertilizers and pesticides, enhanced varieties and transgenic plants, and plant-growth promoting (PGP) microorganisms capable of antagonizing phytopathogens (Compant et al., 2005; Roriz et al., 2020; Al-Tammar and Khalifa, 2022). Currently, a total of 59 landraces of native maize have been described only in México (Hellin et al., 2014). However, relatively few studies have focused on investigating the bacteria from the microbiomes of native maize landraces (Bodhankar et al., 2017; Van Deynze et al., 2018; Chavéz-Díaz et al., 2022; Lund et al., 2022). However, they may offer an opportunity to expand the microbial options for new bioinoculants and SynCom.

In this study, nine endophytic PGP and fungal antagonist bacteria previously isolated from the endosphere of the Jala landrace maize were incorporated into the design of three SynCom (SynCom 1, 2, and 3). Following compatibility tests among strains, the PGP, extracellular enzyme production, the activity of the enzyme phenylalanine ammonia-lyase (PAL), and antifungal *in vitro* capacities of SynCom were evaluated. All SynCom presented relevant phenotypic traits, but SynCom 3 stood out. Plant growth-promoting gnotobiotic and greenhouse assays performed with Conejo landrace seedlings revealed that SynCom 3 significantly increased the length and dry weight of plant shoots and roots compared to the uninoculated control. In addition, the open field experiment performed with hybrid CPL9105W maize inoculated with SynCom 3 significantly increased the maize grain yield and moderately decreased the incidence of fungal rust. This is the first study exploring the potential of endophytic strains of native Mexican maize breeds to formulate synthetic communities and implement them for the improvement of other native maize and improved hybrid maize. This underscores the significance of utilizing these native resources in the rational design of SynCom to foster sustainable agriculture practices.

## 2. Materials and methods

### 2.1. Biological materials

Most of the PGP bacterial strains used in this study were previously isolated from the maize endosphere (steam, root, and seed) of the Jala landrace (Rios-Galicia et al., 2021). *Burkholderia* sp. Z1AL11, *Pantoea* sp. E2AD2, and *P. ananatis* E2HD8 were isolated from a second sampling (Table 1). The strains stored at−70°C were recovered in a solid Luria Bertani (LB) medium and incubated at 28°C for 48 h.

**Table 1:** Plant growth-promoting bacteria used for the assembly of synthetic communities (SynCom).

**Strain (GenBank accession number)**	**Source/habitat**	**Relevant phenotype**
*Burkholderia* sp. Z1AL11 (OQ600603)	Jala landrace/Root endophyte	Phosphate solubilization, IAA, BNF, metallophores, biocontrol against *Fusarium oxysporum, Pestalotia* sp., *Curvularia* sp.
*Achromobacter xylosoxidans* Z2K8 (MK855127)	Jala landrace/Root endophyte	Metallophores, phosphate solubilization, IAA
*Pseudomonas protegens* E2HL9 (MK855122)	Jala landrace/Seed endophyte	Phosphate solubilization, IAA, biocontrol against *Fusarium oxysporum*
*Klebsiella variicola* R3J3HD7 (MK855126)	Jala landrace/Rhizosphere	BNF, phosphate solubilization, IAA, metallophores
*Kosakonia pseudosacchari* Z2WD1 (MK855128)	Jala landrace/Root endophyte	Phosphate solubilization, IAA, metallophores
*Phytobacter diazotrophicus* Z2WL1 (MK855129)	Jala landrace/Root endophyte	IAA production, phosphate solubilization, metallophores
*Pseudomonas protegens* E1BL2 (MK855121)	Jala landrace/Seed endophyte	ACC deaminase activity, IAA, biocontrol against *Fusarium oxysporum, Pestalotia* sp., *Curvularia* sp., *Colletotrichum* sp., *Helminthosporium* sp.
*Pantoea* sp. E2AD2 (OQ600617)	Jala landrace/Seed endophyte	Metallophore production, phosphate solubilization, IAA
*Pantoea ananatis* E2HD8 (OQ606169)	Jala landrace/Seed endophyte	IAA production, phosphate solubilization, metallophores

IAA, indole acetic acid; BNF, biological nitrogen fixation; ACC, 1-aminocyclopropane-1-carboxylic acid.

### 2.2. Compatibility assay

Compatibility was determined by simultaneous inhibition tests to recognize antagonistic interactions among the strains. Overnight cultures of the nine strains were adjusted to an OD_600nm_ of 0.8. A total of 0.1 mL of the suspension was inoculated and distributed on the surface of a Müeller-Hinton (MH) medium. The plates were incubated at 28°C for 48 h. Following the incubation, agar punches were placed on the surface of the MH medium that had previously been massively inoculated with each strain. These plates were pre-incubated at 4°C for 2 h to promote the diffusion of metabolites in the agar and subsequently incubated at 28°C for 24–48 h (Pérez et al., 2014). The inhibition halos represented a negative interaction between each pair of strains. The inhibition pattern among the strains was an important criterion to formulate the SynCom used as bioinoculants.

### 2.3. SynCom formulation

To create the SynCom formulation, the complementary activities of its constituent strains were deemed crucial. This involved ensuring that pairs of strains that demonstrated inhibition in previous tests (2.2) were not included in the same formulation to ensure compatibility. The final SynCom contained strains with complementary phenotypic traits, including nitrogen fixation capability, phosphate solubilization, metallophore production, indoleacetic acid (IAA) production, and ACC deaminase and antifungal activity. Each bacteria strain was grown individually in LB liquid media to the exponential phase. A viable account was carried out to be able to adjust the population density of each bacteria in the formulation at 1.4 × 10^7^ CFU/mL for SynCom 1.2 × 10^7^ CFU/mL for SynCom 2, and 1.4 × 10^7^ CFU/mL for SynCom 3, so that when mixed in a single liquid formulation in LB media, each SynCom has a final density of 1 × 10^8^ CFU/mL, and for subsequent analysis, aliquots of this bacterial mixtures were taken (Armanhi et al., 2018; Rios-Galicia et al., 2021).

### 2.4. Biological nitrogen fixation

The nitrogenase activity was indirectly estimated with an acetylene reduction assay using gas chromatography (Hardy et al., 1968). The axenic bacterial strains and SynCom were grown in sealed bottles containing 5 mL of a semisolid BMGM medium with 50 μL of bacterial suspension adjusted to a final 1x10^8^ CFU/mL concentration. The bottles were sealed and incubated for 48 h at 28°C; *K. variicola* 6A3 was used as a positive control (Rios-Galicia et al., 2021). A volume of 400 μL of the gas contained in the vial was replaced by the same volume of acetylene gas and incubated for up to 6 h, after which the atmosphere of the vial was analyzed by gas chromatography in a Perkin Elmer, Inc. Clarus 580^®^ Ethylene production and residual acetylene (nmol h^−1^) were estimated by integrating the area under the curve.

### 2.5. Phosphate solubilization

The semiquantitative inorganic phosphate solubilization of each axenic bacteria and SynCom were evaluated in plates containing NBRIP medium [10 g/L glucose, 5 g/L Ca_3_(PO_4_)_2_, 5 g/L MgCl_2_•6H_2_O, 0.25 g/L MgSO_4_•6H_2_O, 0.2 g/L KCl, 0.1 g/L (NH_4_)_2_SO_4_ and 16 g/L agar] (Nautiyal, 1999). After 3–5 days of incubation, the solubilization halos around the colonies were measured, and the solubilization indices were reported as the products of the dividing halo and colony diameters (Rashid et al., 2004). The phosphate solubilization by bacteria was quantitatively evaluated in 10 mL of NBRIP liquid medium supplemented with 0.5% hydroxyapatite inoculated 0.1 mL of the adjusted suspension of each SynCom. After an incubation period at 28°C for 5 days, 0.2 mL of aliquot was taken and centrifuged at 13,000 rpm for 10 min. In total, 64 μL of the supernatant was taken and mixed with 16 μL of 0.01 M NaMoO_4_, 80 μL of 0.1 M ascorbic acid, and 40 μL of 5% acetic acid. Absorbance was read at 580 nm (He and Honeycutt, 2005; Bashan et al., 2013).

### 2.6. Indoleacetic acid production

The indoleacetic acid (IAA) production of axenic bacteria and SynCom was evaluated using the colorimetric method employing a Salkowski reagent (0.5 M FeCl_3_ and 35% HClO_4_), which reacts with the indole ring of several related compounds giving it a reddish color. A standard curve of IAA was produced at concentrations ranging from 2 and 200 μg/mL using the Salkowski technique and read at 530 nm (Gordon and Weber, 1951; Szkop et al., 2012).

### 2.7. Metallophore production

The semiquantitative production of metallophores was determined in Chrome Azurol S medium (CAS) plates supplemented with different ion solutions for every metallophore in particular Fe_2_(SO_4_)_3_, CuSO_4_, Na_2_MoO_4_, NaVO_3_, CaSO_4_, MgSO_4_, and ZnSO_4_ (Rios-Galicia et al., 2021). Spots of 5 μL of each axenic bacteria and SynCom were placed on a medium surface, and the plates were incubated at 28°C for 48 h. The yellow halos around the colonies indicated the production of the chelating metallophores of corresponding metal ions. The chelation indices were reported as the products of the dividing halo and colony diameters (Payne, 1994). The metallophores were quantitatively estimated in a liquid CAS medium. The metallophore concentration was proportional to the reduction in blue color intensity produced by removing metals from the chelation CAS-ion complex (Jikare and Chavan, 2013). Bacterial SynCom was grown in a flask containing 10 mL of 10 g/L peptone and 5 g/L NaCl for 48 h at 28°C. After incubation, 0.1 mL aliquots were centrifuged at 13,000 rpm for 15 min. A total of 50 μL of supernatant were added and mixed with 50 μL of CAS solution in a 96-well plate, and the mixture was left to react for 20 min at 25°C. Subsequently, the absorbance at 630 nm was read. An uninoculated medium was used as a control. The chelation percentages were calculated using the following formula:


$$\begin{array}{l} \text{Chelation percentage}=\left[\left(\text{AR}-\text{AS}\right)/\text{AR}\right]\times \;100 \end{array}$$

where:

AR = Absorbance of the reference (CAS medium without inoculation)

AS = Absorbance of the test sample (CAS medium with SynCom inoculation)

### 2.8. Aminocyclopropane-1-carboxylate (ACC) deaminase production

The production of the ACC deaminase enzyme was indirectly determined by the bacterial population growth in LGI culture medium (50 g/L sucrose, 0.01 g/L FeCl_3_•6H_2_O, 0.8 g/L K_2_HPO_4_, 0.2 g/L MgSO_4_•7H_2_O, and 0.002 g/L Na_2_MoO_4_•2H_2_O) supplemented with 1 g/L ACC as the sole source of nitrogen. A total of 180 μL of the liquid LGI culture supplemented with 1 g/L of ACC medium were inoculated with 20 μL of the suspension of each SynCom (0.04 OD_600nm_) in 96-well plates (Johnston-Monje and Raizada, 2011). The plates were incubated at 28°C, and DO600 was determined every 24 h. The negative control was prepared with an LGI medium without ACC.

### 2.9. Growth inhibition of fungal phytopathogens

The antagonist effect of the axenic bacteria and SynCom were evaluated against several phytopathogenic fungal strains isolated from plant tissues with symptoms including *Fusarium oxysporum* (wilting), *Pestalotia* sp. (ear rot), *Curvularia* sp. (leaf spot), *Colletotrichum* sp. (anthracnose), *Helminthosporium* sp. (leaf blight). A 0.8 OD_600_ streak of each SynCom suspension was placed at the center of the plate, and an agar fragment containing mycelial fungal growth was placed on each side. The plates were incubated at 28°C for 5 days (El-Sayed and Edrees, 2014). The percentage of radial growth inhibition (PRGI) was obtained with the following formula:


$$\begin{array}{l} \text{PRGI}=\left(\text{R}1-\text{R}2\right)/\text{R}1\times \;100 \end{array}$$

where:

R1 = Major radius of phytopathogen growth

R2 = Minor radius of inhibition of phytopathogen growth

A commercial aqueous solution of 400 g/100 L copper chloride oxide (CUPRAVIT^®^, Bayer) was used as a positive control for the inhibition.

### 2.10. PAL activity determination for ISR

Germinated fungus-free seeds were placed in 100 g sterile, moistened vermiculite bags. The bags were kept at room temperature for 12 h in light and 12 h in darkness. A total of 5 mL of SynCom or control treatments adjusted to OD_600nm_ of 1 were placed on the base of the shoot on the substrate line. The plants were sampled at vegetative stages VE, V1, and V2 (3, 9, and 12 days, respectively) to determine their height, aerial weight, and root weight. The shoot and leaves were taken separately from the root from these samples and frozen at −20°C until use. To obtain the raw enzyme extracts, the plant tissues were macerated with a lysis solution containing 1% SDS, 3% PVP, and 0.037% EDTA in a ratio of 1:10 P/V until a homogeneous solution was obtained. The tissues were lysed through ten cycles of vortex mixing 1 mL of homogenized tissues and 0.5 g of glass beads in 2 mL cryotubes for 1 min before being placed in an ice bath for 1 min. The lysate was centrifuged at 12,000 rpm at 4°C for 10 min, and the supernatant or cell-free extract was collected. The enzymatic activity of phenylalanine ammonium lyase (PAL) was determined with 200 μL of the cell-free extract and 1,355 μL of 5 mM phenylalanine as a substrate in a 5 mM sodium borate buffer solution having a pH of 8. The mixture was stirred and incubated for 45 min at 25°C. After the incubation, the reaction was stopped with 235 μL of 10% trichloroacetic acid in an ice bath. The samples were read at 290 nm. The PAL unit was defined as the amount of enzyme that allowed the formation of 1 μmol/mL·min of trans-cinnamic acid (Rosler et al., 1997). The Bradford method determined the protein concentration (Bradford, 1976). The specific activity was determined by dividing the volumetric activity by the protein concentration (mg/mL).

### 2.11. Determination of the effect of the inoculation of bacterial SynCom in maize plants

Once the phenotypic characterization tests of the proposed inoculants had been carried out, the effects of each SynCom on maize plants were evaluated.

### 2.12. Seed preparation for gnotobiotic, greenhouse, and open-field PGP assays

The maize seeds were rinsed superficially with distilled water and stirred with deionized water for 2 h at room temperature. The seeds were disinfected by a thermal shock in hot water at 50°C for 10 min followed by 10°C for 5 min in cold water. Subsequently, the seeds were washed with 0.3% HgCl_2_ and rinsed with sterile distilled water ten times. Later, some seeds were placed on a plate with LB agar to verify their proper disinfection (Rios-Galicia et al., 2021).

### 2.13. Preparation of inoculum

For *in planta* gnotobiotic and greenhouse assays, each SynCom and isolated bacterial species used as controls were adjusted to OD_600nm_ of 1. In total, 1 mL of this suspension was then added to each germinated seed.

### 2.14. PGP of maize in gnotobiotic tests

The seeds germinating for 3 days were placed in tubes containing 20 mL of a semi-solid Murashige & Skoog medium (MS) and a suspension of 1 mL of each SynCom; the bacteria were adjusted to OD_600nm_ of 1. The plants inoculated with all the treatments in the mixture were kept at 28°C in darkness for five days and subsequently transferred to a lighting incubator at a room temperature of 24°C, with a photoperiodic lighting of 16 h of light and 8 h of darkness for 20 days (Rekha et al., 2007). The plants were removed from the agar, and the fresh weights and length of the roots and shoots were determined.

The treatments were as follows: seeds inoculated with SynCom 1, SynCom 2, SynCom 3, *P. protegens* E1BL2 (positive control), and sterile water (negative control).

### 2.15. PGP of maize in greenhouse tests

Groups of six seeds were inoculated with 1 mL per seed of each treatment, germinated for 3 days, and incubated for 24 h. The seeds were sown in plastic pots with sterile vermiculite and irrigated every third day with a Hoagland nutrient solution to avoid overhydration, dehydration, pests, or pH alterations (Rios-Galicia et al., 2021). Periodic observations of plant conditions were performed over 60 days.

The treatments are as follows: seeds inoculated with SynCom 1, SynCom 2, SynCom 3, *P. protegens* E1BL2 (positive control), and sterile water (negative control). The shoot and root lengths and dry weights were determined at the end of the 60 days. The dry weights were determined by oven drying at 55°C until a constant weight was reached.

### 2.16. PGP of maize in open field tests

The field test evaluated the effect of the inoculation of SynCom on the maize crop yield and rust incidence. The experiment was performed in the experimental fields of the Innovation and Technological Development Center Mezquital Valley, located in the Cinta Larga municipality of Mixquiahula Hidalgo, Mexico (20.192457,−99.243521). The sowing was performed during the third week of May 2019. Commercial hybrid seeds CPL9105W from CROPLAN Genetics^®^ were used for the test.

The seeds were washed with tap water to remove the insecticides and fungicides until the wash water had no color. They were then dried with absorbent paper, placed in a plastic bag, and inoculated using a suspension of SynCom and *P. protegens* E1BL2 used as a PGP positive control at a concentration of 1 × 10^8^ CFU/mL. The bacteria were obtained from a culture grown for 48 h in an LB broth. Carboxymethylcellulose was used as an adherent with 30 g/kg of seeds. The negative controls with distilled water and the LB media were incorporated into the experiment.

The evaluation was completely randomized over five repetitions in experimental plots 5 m long with four furrows and 80 cm of separation between each one. Three seeds were sown and separated by a distance of 17.5 cm. Irrigation was applied monthly. The herbicides Marvel^®^ (3,6-dichloro-2-methoxy benzoic acid and 6-chloro-N-ethyl-N-isopropyl-1,3,5-triazine-2,4-diamine) were applied at a dose of 2 L/ha after the first irrigation. At 30 days, the smaller plants were removed to obtain one plant per hole and a uniform population of 112 plants per row, equivalent to a population density of 72,500 plants/ha. Three applications of the SynCom and control were made monthly per treatment, and 2.5 L of the formulated SynCom and control were sprayed 25 days after the sowing.

To determine the yield, the ears of the plants in the central rows were counted in each plot. A total of 22 ears of each treatment were taken randomly, weighed, and shelled. The kernels were also weighed. Seed moisture was determined using a brand grain moisture tester (John Deere, IL, USA). The shelled factor (SF) was calculated using the following formula:


$$\begin{array}{l} \text{SF}=\left(\text{Weight of kernels from }22\text{ ears}/\text{Total weight of }22\text{ ears}\right). \end{array}$$

With this data, the yield (Y) in Ton/ha was calculated using the following formula:


$$\begin{array}{l} \text{Y}=\left[\left(\text{W}/22\text{ears}\right)\left(\text{T}\right)\left(\text{HPG}/\;86\right)\left(\text{SF}\right)\left(1000/\text{G}\right)\right]/1000 \end{array}$$

where:

W = Average weight (kg) of 22 ears

T = Total number of ears in central rows

HPG = Humidity percentage of kernels

86 = Standardized yield factor at 14% humidity

SF = Shelled factor

G = Furrow width (0.8 m).

The rust incidence percentage in each plot was also evaluated at 90 days after sowing using the Peterson scale (Peterson et al., 1948).

### 2.17. Statistical analysis

All phenotypic experiments were assayed in triplicate and repeated at least two times, for *in planta* gnotobiotic and greenhouse were assayed with six plants per treatment and repeated two times. An ANOVA was carried out for the phenotypic experiments. The means of each treatment were compared using Duncan's multiple range test at a 0.05 level. The data are shown as the means and their standard errors. The plant growth data were analyzed using a completely randomized design, and Tukey's Honest Significance Differences test compared the means. Differences were considered significant at a *P*-value of < 0.05. Statistical analyses were performed with GraphPad Prism version 9.00 (GraphPad Software, La Jolla California, USA).

## 3. Results

### 3.1. Compatibility assay and SynCom design

**Figure 1:**
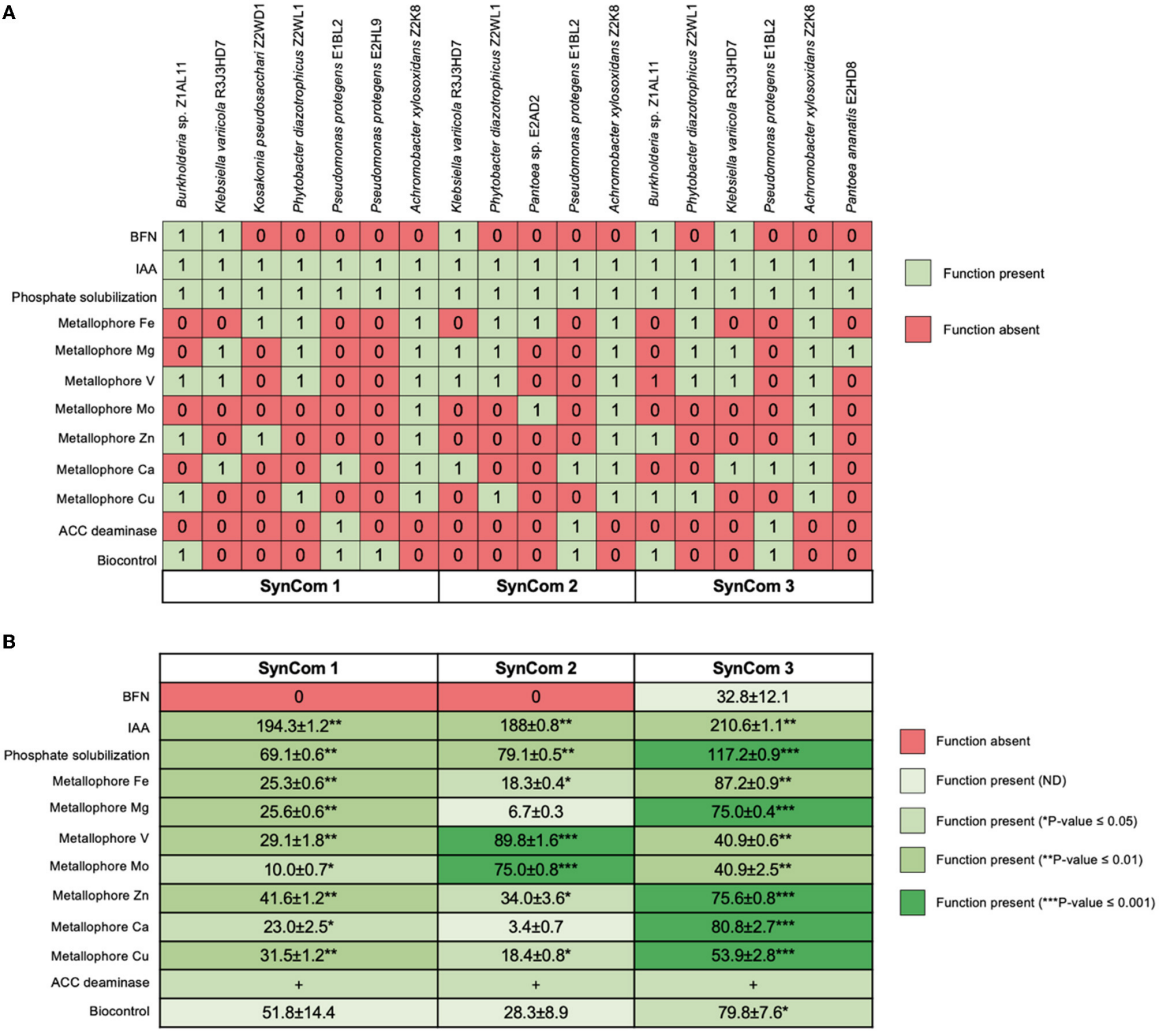
Functional traits of Jala maize endophytes individually and in the synthetic microbial communities (SynCom). The growth promotion and biocontrol **(A)** capabilities of each of the studied isolates are shown and compared with **(B)** the formulated SynCom. The colors represent the absence and presence of these activities as well as statistically significant differences according to Duncan's test (^*^P ≤ 0.05, ^**^P ≤ 0.01, and ^***^P ≤ 0.001).

Antagonistic interactions were only detected for *Pantoea* sp. E2AD2 on *Burkholderia* sp. Z1AL11, *P. protegens* E2HL9, *P. ananatis* E2HD8, and *K. pseudosacchari* Z2WD1; and for *P. ananatis* E2HD8 on *P. protegens* E2HL9 and *K. pseudosacchari* Z2WD1. The SynCom design included bacterial strains with partially redundant and complementary PGP features, but antagonistic strains were not included (Figure 1). The final formulations were SynCom 1: *A. xylosoxidans* Z2K8, *Burkholderia* sp. Z1AL11, *K. variicola* R3J3HD7, *K. pseudosacchari* Z2WD1, *P. diazotrophicus* Z2WL1, *P. protegens* E1BL2, *P. protegens* E2HL9; SynCom 2: A. xylosoxidans Z2K8, K. variicola R3J3HD7, P. diazotrophicus Z2WL1, *Pantoea* sp. E2AD2, *P. protegens* E1BL2; SynCom 3: *A. xylosoxidans* Z2K8, *Burkholderia* sp. Z1AL11, *K. variicola* R3J3HD7, *P. diazotrophicus* Z2WL1, *P. ananatis* E2HD8, *P. protegens* E1BL2. Each SynCom contained a population of 1 × 10^8^ CFU/mL; each bacterial species was in the same cellular population. These suspensions were used for the *in vitro* and *in planta* tests.

### 3.2. PGP traits of mixed bacterial inoculants

The *in vitro* phenotypic PGP tests of the axenic bacteria and SynCom are shown in Table 2. The semiquantitative phosphate solubilization test revealed no differences between the SynCom and control strains, but the quantitative assay differences were evident. SynCom 3 accumulated more soluble orthophosphate (117 μg/mL) in 96 h than the other axenic bacteria, including *Pantoea* sp. E2AD2 (29 μg/mL), SynCom 1 (69 μg/mL), and SynCom2 (79 μg/mL).

**Table 2:** Features of plant growth-promoting bacteria in isolates and SynCom.

**Strain and SynCom**	**BNF (nmol of ethylene h^−1^)**	**Phosphate solubilization**		**IAA (μg/mL)**	**Metallophores**														**ACC deaminase**
					**Fe**		**Mg**		**V**		**Mo**		**Zn**		**Ca**		**Cu**		
		**Semiquantitative (mm)**	**Quantitative (**μ**g/mL)**		**Semiquantitative (mm)**	**Quantitative (%)**	**Semiquantitative (mm)**	**Quantitative (%)**	**Semiquantitative (mm)**	**Quantitative (%)**	**Semiquantitative (mm)**	**Quantitative (%)**	**Semiquantitative (mm)**	**Quantitative (%)**	**Semiquantitative (mm)**	**Quantitative (%)**	**Semiquantitative (mm)**	**Quantitative (%)**	
*Burkholderia* sp. Z1AL11	16.6 ± 3.1	2.0 ± 0.3	10.8 ± 0.3	8.95 ± 0.2	4 ± 0.8	-	-	-	4 ± 0.4	5.8 ± 0.6	3 ± 0.7	-	4 ± 0.6	5.3 ± 0.6	3 ± 0.4	-	3 ± 0.4	11.2 ± 0.9	-
*Achromobacter xylosoxidans* Z2K8	-	1 ± 0.2	-	13.8 ± 0.6	2 ± 0.3	15.8 ± 0.7	2 ± 0.2	10 ± 0.4	2 ± 0.6	18.9 ± 0.5^*^	3 ± 0.6	10.1 ± 0.4^*^	4 ± 0.8	34 ± 0.9^*^	2 ± 0.4	18.8 ± 1.6^*^	2 ± 0.6	19.1 ± 1.2^*^	-
*Pseudomonas protegens* E2HL9	-	1.8 ± 0.1	10.7 ± 0.3	3.12 ± 0.3	3 ± 0.4	-	1 ± 0.5	-	3 ± 0.4	-	2 ± 0.4	-	4 ± 0.6	-	2 ± 0.5	-	2 ± 0.3	-	-
*Klebsiella variicola* R3J3HD7	28.5 ± 8.6	2.5 ± 0.3	11 ± 0.2	7.46 ± 0.4	1 ± 0.7	-	-	17.8 ± 0.6^*^	1 ± 0.8	7.9 ± 0.9	1 ± 0.2	-	2 ± 0.4	-	2 ± 0.3	2.7 ± 1.1	-	-	-
*Kosakonia pseudosacchari* Z2WD1	-	2.1 ± 0.4	14.2 ± 0.3	3.73 ± 0.2	1 ± 0.4	5.7 ± 0.3	1 ± 0.2	-	1 ± 0.4	-	1 ± 0.2	-	2 ± 0.6	4.6 ± 0.2	1 ± 0.8	-	1 ± 0.2	-	-
*Phytobacter diazotrophicus* Z2WL1	-	1 ± 0.3	-	48.1 ± 0.9^*^	2 ± 0.5	12.7 ± 0.7^*^	1 ± 0.4	23.5 ± 0.4^*^	2 ± 0.5	4.8 ± 0.7	2 ± 0.5	-	4 ± 0.9	-	-	-	-	23.6 ± 0.5^*^	-
*Pseudomonas protegens* E1BL2	-	1 ± 0.2	-	4.33 ± 0.4	3 ± 0.3	-	3 ± 0.5	-	3 ± 0.4	-	4 ± 0.4	-	4 ± 0.6	-	3 ± 0.5	3.3 ± 0.6	3 ± 0.7	-	+
*Pantoea* sp. E2AD2	-	2.8 ± 0.5	29.3 ± 0.5^*^	32.1 ± 0.5^*^	4 ± 0.8	3.6 ± 0.6	3 ± 0.3	-	3 ± 0.6	-	3 ± 0.4	3.9 ± 0.6	3 ± 0.4	-	3 ± 0.3	-	3 ± 0.6	-	-
*Pantoea ananatis* E2HD8	-	1.2 ± 0.1	10.6 ± 0.3	51.7 ± 0.9^*^	1 ± 0.3	-	2 ± 0.1	5.7 ± 0.6	-	-	1 ± 0.5	-	1 ± 0.3	-	-	-	1 ± 0.3	8.4 ± 0.6	-
SynCom 1	-	2.4 ± 0.4	69.1 ± 0.6^**^	194.3 ± 1.2^**^	4 ± 0.7	25.3 ± 0.6^**^	2 ± 0.3	25.6 ± 0.6^**^	3 ± 0.5	29.1 ± 1.8^**^	2 ± 0.4	10 ± 0.7^*^	2 ± 0.4	41.6 ± 1.2^**^	2 ± 0.4	23 ± 2.5^*^	3 ± 0.3	31.5 ± 1.2^**^	+
SynCom 2	-	2.6 ± 0.4	79.1 ± 0.5^**^	188 ± 0.8^**^	3 ± 0.5	18.3 ± 0.4^*^	1 ± 0.2	6.7 ± 0.3	3 ± 0.6	89.8 ± 1.6^***^	2 ± 0.8	75 ± 0.8^***^	2 ± 0.8	34 ± 3.6^*^	3 ± 0.5	3.4 ± 0.7	2 ± 0.6	18.4 ± 0.8^*^	+
SynCom 3	32.8 ± 12.1	3 ± 0.3	117.2 ± 0.9^***^	210.6 ± 1.1^**^	2 ± 0.6	87.2 ± 0.9^***^	3 ± 0.1	75.0 ± 1.4^***^	3 ± 0.6	40.9 ± 0.6^**^	3 ± 0.9	40.9 ± 2.5^**^	3 ± 0.5	75.6 ± 0.8^***^	2 ± 0.4	80.8 ± 2.7^***^	4 ± 1.2	53.9 ± 2.8^***^	+

The features of bacterial isolates and SynCom were compared using Duncan's test (^*^p ≤ 0.05, ^**^p ≤ 0.01, and ^***^p ≤ 0.001). The values are expressed as the mean ± SD (n = 3). IAA, indole acetic acid; BNF, biological nitrogen fixation; ACC, 1-aminocyclopropane-1-carboxylic acid; Fe, iron; Mg, magnesium; V, vanadium; Mo, molybdenum; Zn, zinc; Ca, calcium; Cu, copper.

All axenic bacteria produced between 3.1 and 51.7 μg/mL of IAA in a culture medium supplemented with 51.7 μg/mL tryptophan. *P. ananatis* E2HD8 was the strain that produced the highest amount of IAA. All synthetic communities produced from 188 to 210.6 μg/mL of IAA, but SynCom 3 was the most efficient.

Aminocyclopropane-1-carboxylate (ACC) deaminase production was only detected in *P. protegens* E1BL2; the other *P. protegens* E2HL9 did not express this enzymatic activity. All SynCom contained the *P. protegens* E2HL9 strain and expressed the activity. Different profiles of metallophores for different ions (Co^2+^, Ca^2+^, Mg^2+^, Zn^2+^, Fe^3+^, Mo^2+^, and V^5+^) were observed in both the bacteria and SynCom. Nitrogen fixation was only detected in *Burkholderia* sp. Z1AL11 and *K. variicola* R3J3HD7. Although all SynCom had at least one diazotrophic bacterium, only SynCom 3 exhibited evident growth in the BMGM media and reduced acetylene allowing 32.8 ± 12.1 nmol of ethylene h^−1^.

### 3.3. Growth inhibition of fungal phytopathogens

**Figure 2:**
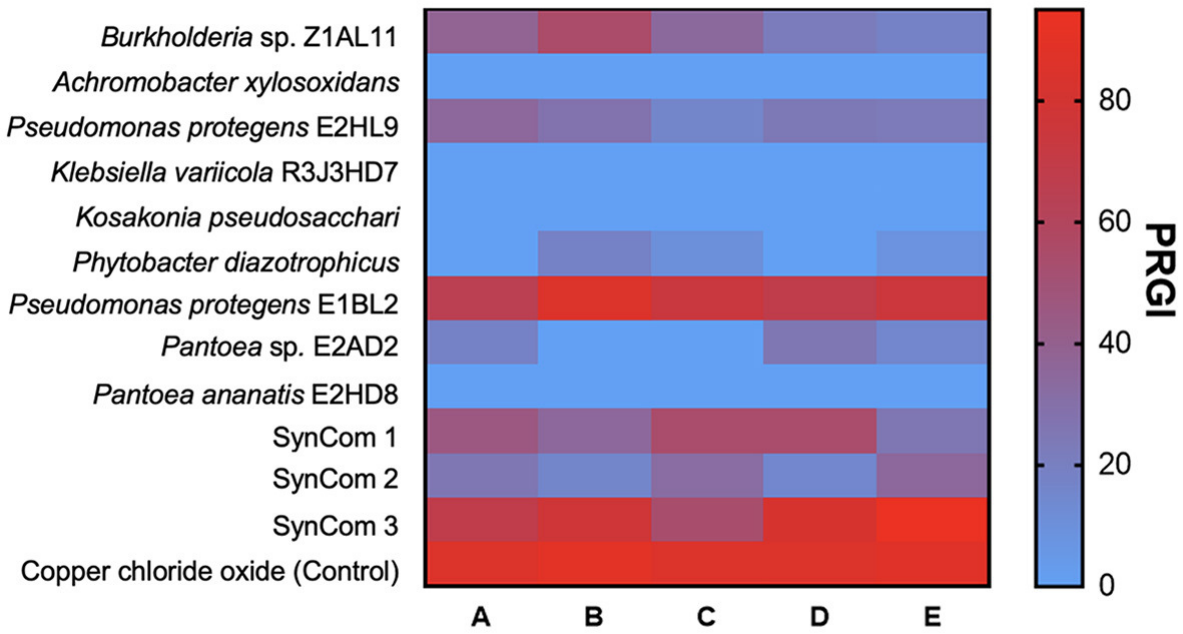
Heat map of the fungal radial growth inhibition by endophytic bacteria. **(A)** *Fusarium oxysporum*, **(B)** *Pestalotia* sp., **(C)** *Curvularia* sp., **(D)** *Colletotrichum* sp., and **(E)** *Helminthosporium* sp.

The inhibition of fungal growth by PGPB and SynCom is shown in Figure 2. *P. protegens* E1BL2 and SynCom 3 exhibited the highest inhibition percentages reaching 80% of inhibition. *Pestalotia* sp. and *Helminthosporium* sp. were hardly inhibited by *P. protegens* E1BL2 and SynCom 3, respectively.

### 3.4. PAL activity determination for ISR

**Figure 3:**
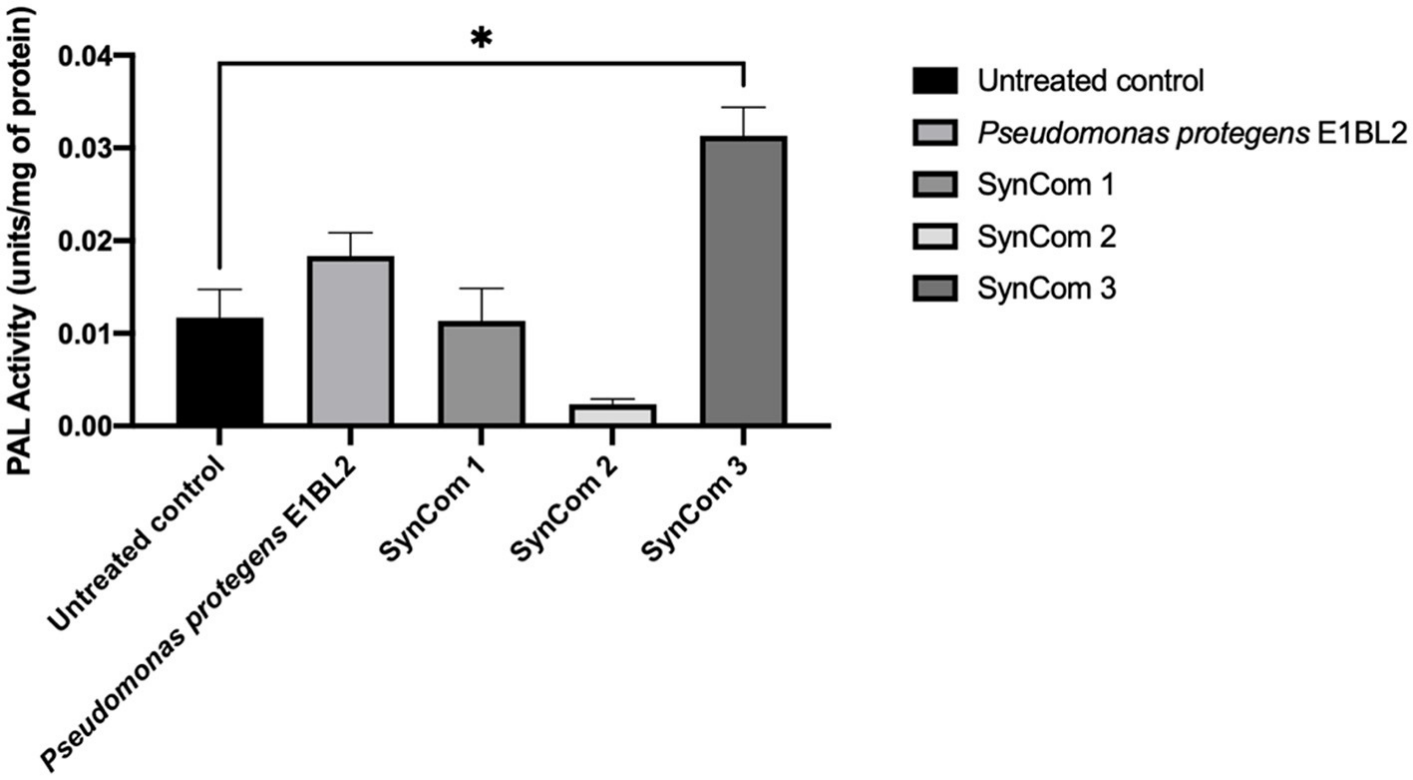
Enzymatic activity of maize roots' phenylalanine ammonium lyase (PAL) treated with inoculants; the *P. protegens* E1BL2 strain was used as a control. The PAL activity of inoculated and uninoculated plants was compared using Tukey's test (*p ≤ 0.05).

The treatments of the plants with *P. protegens* E1BL2 and SynCom 3 increased the PAL enzymatic activity of the corn root tissues. However, as shown in Figure 3, only the effect of SynCom 3 was statistically significant as it tripled the activity of the enzyme of the untreated control.

### 3.5. *In planta* assay in gnotobiotic and greenhouse systems

**Figure 4:**
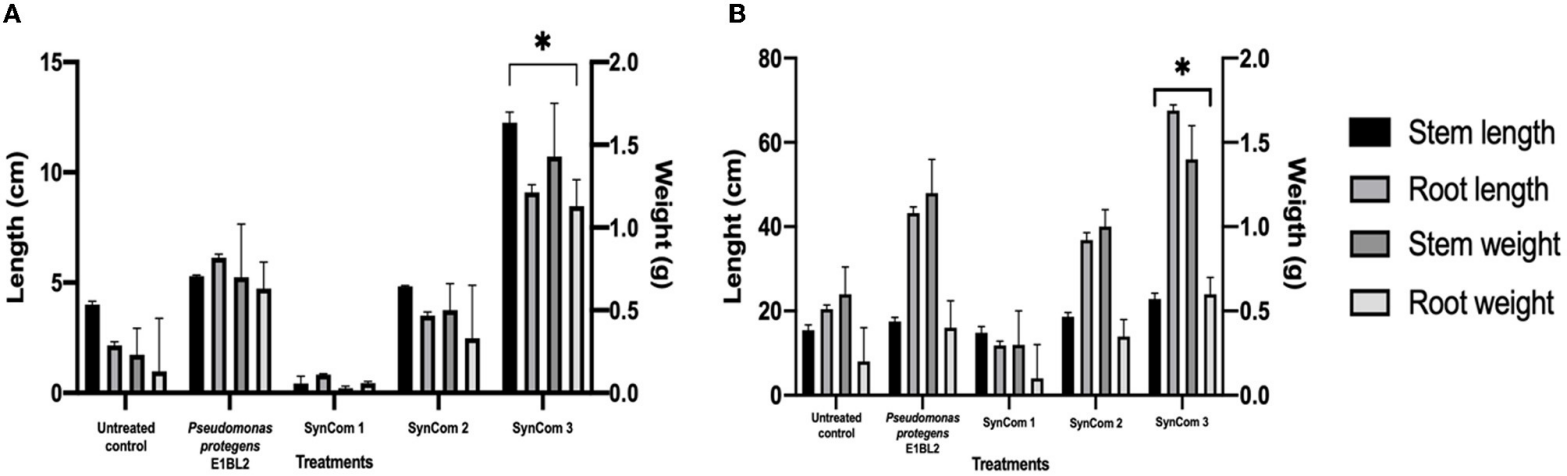
Effect of the inoculation of SynCom on the shoot, root length, and weight of the Conejo landrace. **(A)** Application of SynCom bioinoculants on the gnotobiotic system and **(B)** greenhouse system. Tukey's test compared the lengths and weights of inoculated and uninoculated plants (*p ≤ 0.05).

**Figure 5:**
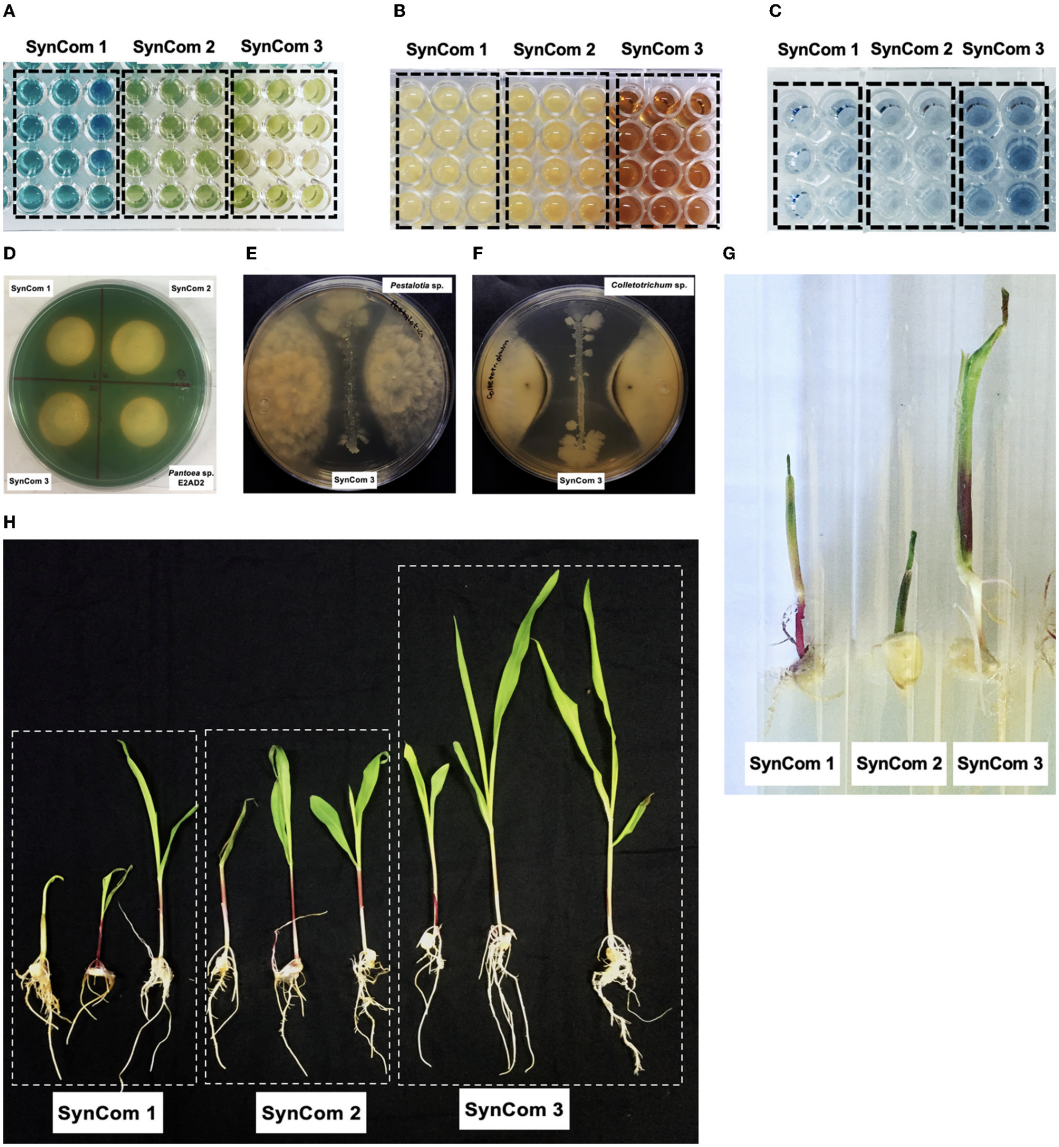
Phenotypic traits of Jala maize SynCom. **(A)** Quantitative metallophore test in a liquid medium, blue colors represent low production, and green and yellow colors represent high production. **(B)** In quantitative IAA determination, yellow colors represent low production, and orange and red colors represent high production. **(C)** In quantitative phosphate solubilization, the intensity of the blue color is proportional to the production of orthophosphate. **(D)** Metallophore production test in plates, the size of the halos was determined to estimate the production of each particular metallophore. **(E)** Inhibition of mycelial growth of *Pestalotia* sp. by SynCom 3. **(F)** Inhibition of mycelial growth of *Colletotrichum* sp. by SynCom 3. **(G)** Plant growth promotion assay in a gnotobiotic system by SynCom. **(H)** Plant growth promotion in a greenhouse system by SynCom.

The effect of SynCom on plant growth in the gnotobiotic system is shown in Figure 4A. The promotion of seedling growth was highlighted by SynCom 3, which promoted the growth of the roots and shoots to sizes 4.2 and 3.0 times greater, respectively, than those of the untreated control. The effect of SynCom on plant growth at the greenhouse level is shown in Figure 4B, with increases of 3.3 and 1.4 times the roots and shoot length and 2.3 and 3 times the dry weights of the shoots and roots, respectively. Examples of *in vitro* PGP traits and *in planta* assays are illustrated in Figure 5.

### 3.6. Field test

**Figure 6:**
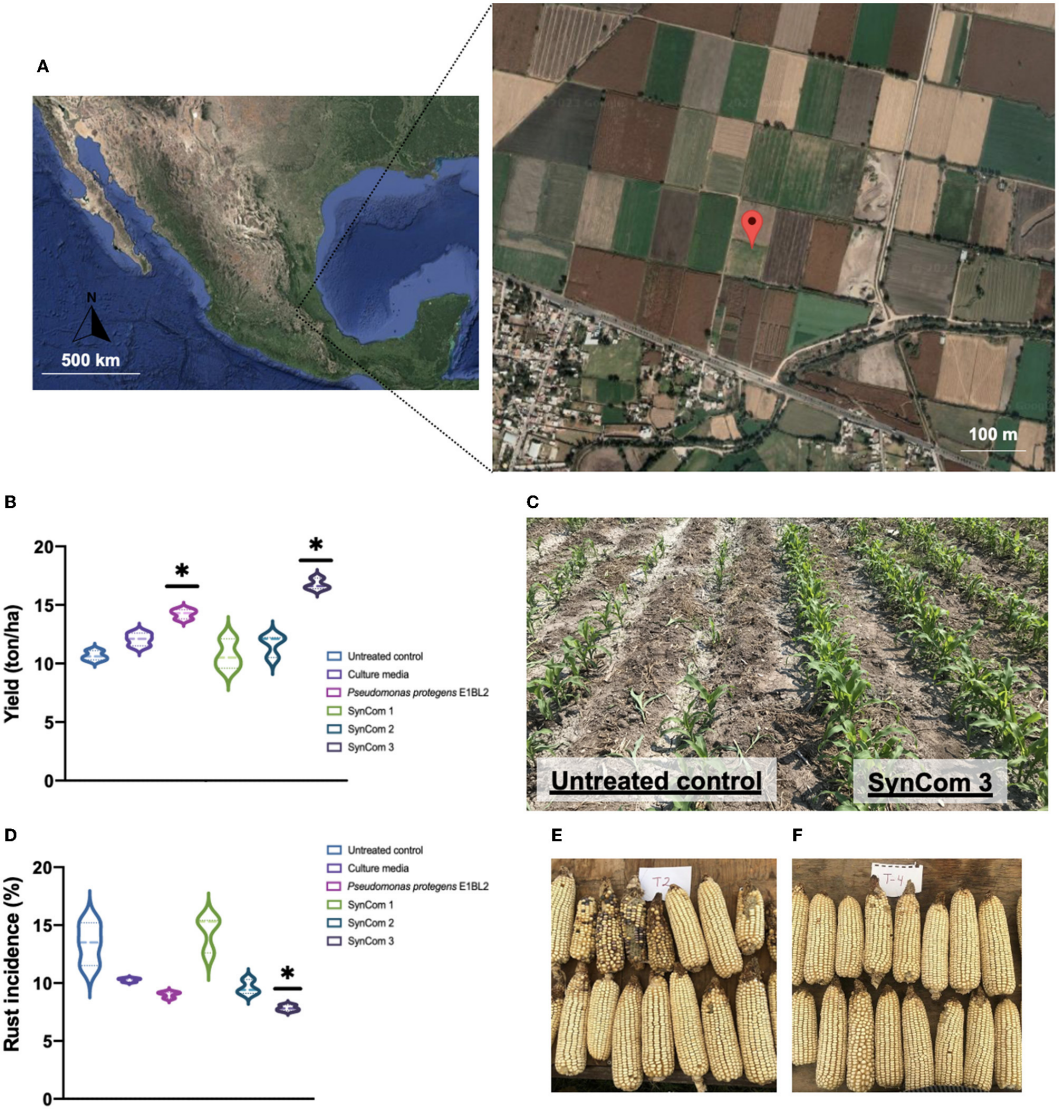
Field test of bacterial SynCom application in maize crop using the commercial hybrid CPL9105W. **(A)** Geographic localization of the open field in Mixquiahula Hidalgo, Mexico (20.192457,−99.243521). **(B)** Bacterial SynCom effect in the crop yield (ton/ha). **(C)** Bacterial SynCom effect in the rust incidence percentage (%). **(D)** A comparison of untreated control plants and SynCom 3 treatment. **(E)** Ears or maize without treatment and **(F)** ears treated with SynCom 3. The test was conducted in the experimental fields from the Innovation and Technological Development Center Mezquital Valley, Mixquiahula Hidalgo, México. Tukey's test compared the yield and rust incidence of inoculated and uninoculated plants (*p ≤ 0.05).

As shown in Figure 6, the productivity yield of the control treatment was 11 Tons/ha, but *P. protegens* E1BL2 and the SynCom 3 treatments resulted in yields of 14 and 16 Tons/ha, respectively. Moreover, the incidence of rust infection showed a significant decrease of 12.5% with the SynCom 3 treatment. SynCom 1 and 2 did not show significant increases in productivity or decreases in rust infection concerning the controls.

## 4. Discussion

The Tehuacán and Balsas Valleys in Mexico are the biological origin of maize and its domestication, artificial selection, and initial diversification that currently include at least 59 landraces of native maize. The PGP and fungal antagonistic bacteria used in this study were isolated from the endosphere of native maize of the Jala landrace, which is grown in the states of Jalisco and Nayarit in Mexico (Rios-Galicia et al., 2021). However, the PGP test was performed on the Conejo maize landrace because it is a tropical strain prone to fast growth, reaches physiological maturity around 90 days, and is adapted to humid coastal climates and dry tropics (Wilkes, 1977). CPL9105W hybrid maize seeds were used for the field trials due to their better adaptation to the field climate and early maturation time.

All SynCom displayed better *in vitro* PGP capabilities than the strains used in individual trials. This synergistic effect mainly observed in maize, rice, and wheat assays confirms the advantages of the design of the three SynCom (Olanrewaju and Babalola, 2019; Ngalimat et al., 2021; Cherif et al., 2022). However, any comparison of the quantitative results obtained in this study with previous data must be made with caution and considering the context because many methods and conditions for PGP and antifungal assays have been reported in the literature.

Phosphorus is an essential component of molecules such as DNA and ATP and it is vital for photosynthesis, energy transfer, and cell reproduction. In addition, phosphorus is necessary for the growth and production of strong and resistant roots, which is important for disease resistance and soil nutrient intake; thus, applying soluble phosphate microorganisms is a cost-effective alternative for producing this mineral (Rawat et al., 2021; Elhaissoufi et al., 2022). Although the strains in this study did not reach the levels of phosphate solubilization of *Serratia* sp. S119 (70 μg/mL) (Ludueña et al., 2018), all the SynCom generated similar or higher yields.

Indoleacetic acid (IAA) is an auxin phytohormone that has complex effects on the growth of different plant organs depending on its concentration, life cycle stage, the affected tissue, and an endogenous or exogenous origin (Duca et al., 2014; Kunkel, 2021). The constant exogenous supply of this hormone in quantities not exceeding 10 nM (growth-inhibiting concentration in many plant species) ensures the development of the roots (Eliasson et al., 1989). The axenic bacterial strains produced IAA similar to those of *Azospirillum brasiliense* strains (21 to 102 μg/mL) (Meza et al., 2015), but all SynCom synthesized significantly higher IAA final concentrations. The explanation for this phenomenon is beyond the scope of this study. Still, it could partly explain the shoot and root growth promotion documented in the trials under gnotobiotic, greenhouse, and open field conditions.

Metallophores, particularly siderophores, are secondary metabolites with metal ion chelating activity secreted by bacteria. In addition, to supply Fe and other essential metals to plants and promote their growth, metallophores provide additional benefits such as limiting the development of some phytopathogen fungi and bacteria and binding and detoxifying toxic heavy metals in heavy metal-contaminated soils (Timofeeva et al., 2022). The production capacity of metallophores is widely distributed among bacteria, and the species used in this study are no exception (McRose et al., 2018). The tests could not discern if the strains produced a single metallophore with several chelating capabilities or if several metallophores with specific affinities were excreted. Still, it seems to be a frequent phenomenon in bacteria (McRose et al., 2018; Reitz, 2022). The SynCom included bacteria species with a vast display of siderophores.

The aminocyclopropane-1-carboxylate (ACC) deamination ability of bacteria provides plants with an additional nitrogen source of ammonium, which plants can also use. Both IAA and ACC can be synthesized by plants and bacteria cells. Under stress conditions, IAA and ACC increase and induce a stress response *via* ethylene, a compound that can inhibit plant growth. The excess ACC is exported to associated bacteria, and ACC deaminase metabolizes to ammonia and a-ketobutirate. A bioinoculant with both characteristics would help regulate the plant synthesis of ethylene under stress conditions (Glick, 2014). SynCom 3 contained bacteria with multiple phenotypic traits probably responsible for increased plant growth observed in the assays. In part, this growth resulted from ethylene levels that have remained low during the plant's life cycle since endogenous IAA synthesis was inactivated thanks to an external supply (Etesami et al., 2014). In addition, the production of the ACC synthase and oxidase enzymes in response to stress is regulated by the intervention of the bacterial deaminase ACC (Glick, 2005; Van de Poel and Van Der Straeten, 2014). Thus, while the entry of exogenous IAA promotes growth and contributes to diminishing its endogenous synthesis, the enzyme ACC-deaminase reduces the impact of the wave of endogenous ethylene synthesis that occurs in response to stress by acting as an exhaust valve that prevents ethylene from reaching harmful levels (Singh et al., 2015; Nascimento et al., 2018; Mou et al., 2020).

All formulated SynCom contained the strain of *K. variicola* R3J3HD7, but only SynCom 3 reduced acetylene and possibly fixed nitrogen *in vitro*. The free-living nitrogen-fixing ability of *K. variicola* has previously been reported (Haahtela et al., 1983; Qin et al., 2014), and its importance in open-field experiments has been recognized (Guerrieri et al., 2021; Kusale et al., 2021). The abundant polysaccharides of *K. variicola* probably generated a physicochemical barrier to molecular oxygen to protect oxygen-sensitive nitrogenase (Fourmond and Léger, 2017; Kubas et al., 2017). In addition, the *Burkholderia* sp. Z1AL11 diazotrophic strain was included in the SynCom 3 design. The nitrogen fixation capability is distributed among some species of the *Burkholderia cepacia* complex (Sandanakirouchenane et al., 2017; Li et al., 2022). The specific contribution to the final nitrogen fixation of each species in the SynCom is unknown, but this PGP feature is essential in the formulation.

SynCom 3 contained *Burkholderia* sp. Z1AL11 and *P. protegens* E2HL9 and exhibited an apparent antagonistic effect on several phytopathogenic fungi *in vitro*, in addition to decreasing the incidence of rust in the open-field experiment. This antifungal effect may have been due to nutrient competition and the production of siderophores and soluble or volatile antifungals (Mannaa and Kim, 2018; Pellicciaro et al., 2021). *P. protegens* produces biocontrol compounds such as 2,4-diacetyl phloroglucinol, pyoluteorin, pyrrolnitrin, phenazines, and hydrogen cyanide (Haas and Keel, 2003; Haas and Défago, 2005; Raaijmakers et al., 2009; Ramette et al., 2011; Zhang et al., 2020). The antifungal ability is also widely distributed among the *Burkholderia* genus (Depoorter et al., 2016; Elshafie and Camele, 2021).

Plants colonized with endophytic bacteria can induce an efficient and rapid systemic resistance to phytopathogens. In the absence of challenge trials with phytopathogen fungi, the PAL activity of SynCom was used as an indicator of this adaptive response. Numerous papers have described this response; for example, *Citrullus lanatus* and *Cucumis sativus* exposed to a non-pathogenic strain of *Colletotrichum magna* showed high levels of lignin deposition, peroxidase activity, and PAL activity and protection against diseases caused by *Colletotrichum orbiculare* and *Fusarium oxysporum* (Fu-kang et al., 2010; Hassan et al., 2014; Elsharkawy et al., 2015). More detailed study is needed, but SynCom 3 decreased the frequency of fungal rust in the open-field experiment.

The set of experiments proposed in this study did not allow us to determine the contribution of each of the bacteria and their phenotypic traits in the observed PGP at the gnotobiotic, greenhouse, or open field levels. However, the rational design of SynCom suggests that it is an efficient strategy that can be evaluated experimentally at various levels. The “Theory of Multiple Mechanisms” could explain the effects on PGP,” where the impact of individual mechanisms, operating simultaneously or consecutively, will result in a more significant effect on the plant (Mendoza and Cruz, 2012). The SynCom are new alternatives for an ecological and environmentally friendly approach as they arise from and are applied to the corn environment, making use of the same natural resources in an optimized manner and considering the plant-microorganisms' holobiont relationship (Chiu and Gilbert, 2015; Sánchez-Cañizares et al., 2017; Cesaro et al., 2021). The increase in grain productivity and reduction of fungal infections, even when modest, offer alternatives to the indiscriminate use of chemical fertilizers and recalcitrant pesticides and should thus certainly be investigated further (Xue et al., 2016; Jat et al., 2021; Kiani et al., 2021).

## 5. Conclusion

The inoculation of a SynCom of growth-promoting bacteria composed of the strains *Burkholderia*. sp. Z1AL11, *A. xylosoxidans* Z2K8, *K. variicola* R3J3HD7, *P. diazotrophicus* Z2WL1, *P. protegens* E1BL2, and *P. ananatis* E2HD8 promoted the growth of Conejo landrace maize plants in gnotobiotic and greenhouse trials. Treatments of maize using this synthetic community also increased productivity and decreased the rust incidence in hybrid CPL9105W crops.

The studied SynCom also inhibited the growth of some phytopathogenic fungi, making it a potential biofertilizer and bio fungicide that could be applied in the field.

Bacterial SynCom promotes the defense activity of plants by providing them with an ISR is one of the strategies that should be used as an alternative to recalcitrant pesticides and environmentally harmful contaminants.

## Data availability statement

The raw data supporting the conclusions of this article will be made available by the authors, without undue reservation.

## Author contributions

ED, JS-A, and BR-G performed the bacterial isolation and characterization and conducted the greenhouse experiments. YM-F provided the seed samples and coordinated the field test. RA-G, LV-T, and CH-R provided the samples and designed and coordinated the study. ED wrote the first draft of the manuscript. ED, JS-A, BR-G, YM-F, RA-G, LV-T, and CH-R contributed to the editing of the manuscript. All authors read and approved the final manuscript.
